# Influence of Dietary Salt Intake on T2D Treatment

**DOI:** 10.3389/fendo.2022.926143

**Published:** 2022-06-15

**Authors:** Li Li, Yuwei Mi, Miao Xu, Liemin Ruan, Jie Sun, Qifa Song

**Affiliations:** ^1^ Endocrinology and Metabolism, Ningbo City First Hospital, Ningbo, China; ^2^ Medical Data Center, Ningbo City First Hospital, Ningbo, China

**Keywords:** dietary, effect, salt intake, treatment, type 2 diabetes

## Abstract

**Backgrounds:**

To what extent patients undergoing long-term T2D treatment are affected by dietary salt intake has not been completely investigated.

**Objectives:**

We aimed to investigate the influence of dietary salt intakes on T2D treatment, including glucose-lowering effect and indices related to T2D progression.

**Methods:**

The study recruited 1090 patients with T2D at Ningbo City First Hospital from January 1, 2018, to December 30, 2021. We compared their one-year follow-up outcomes in terms of fasting blood glucose (FBG), glycated hemoglobin (HbA1c), blood pressure, obesity, and prevalence of retinopathy and neuropathy among groups with different dietary salt intakes.

**Results:**

The 1090 patients consisted of 287(26.3%) decreasing-, 190(17.4%) increasing-, 175(16.0%) steadily low-, 243(22.3%) steadily medium-, and 195(17.9%) steadily high-dietary salt intake patients. Compared to increasing-, steadily medium-, and steadily high-dietary salt intake patients, decreasing and steadily low salt intake led to lower baseline FBG, HbA1c, systolic blood pressure (SBP), BMI, and visceral fat area (VFA) (all p<0.05), to a larger decrease in FBG, HbA1c, SBP, BMI, and VFA after one-year treatment (all p<0.05), as well as to a slightly lower prevalence of retinopathy and a significantly lower prevalence of neuropathy. The steadily low salt patients had lower urine albumin/creatinine ratio (UAR) both at baseline and after treatment. Notably, the fasting insulin in the steadily low salt group was higher than the remaining groups after treatment (p<0.01).

**Conclusions:**

The present study concludes that lowered dietary salt intake benefits T2D treatment in multiple aspects, including main treatment targets such as FBG and HbA1c, and indices reflecting potential complications of T2D, including BMI, VFA, SBP, UAR, retinopathy, and neuropathy.

**Clinical Trial Registration:**

www.ClinicalTrials.gov, identifier: NCT03811470.

## Introduction

Currently, the number of patients with type 2 diabetes (T2D) has been steadily increasing globally. The International Diabetes Federation (IDF) estimated that in 2015, globally, 415 million adults had diabetes mellitus, 90% of which had T2D. A large proportion of T2D patients are afflicted with comorbidities including obesity, hypertension, cardiovascular illness, and kidney impairment (1). T2D is highly linked to unhealthy lifestyles of people, mainly including nutrient intake and physical activity (2). Dietary habits are one primary aspect of lifestyles and linked to T2D, hypertension, and cardiovascular diseases (3, 4). Besides the medication treatment of T2D, lifestyle optimization and education are essential for patients with T2D or showing prediabetic symptoms to achieve beneficial therapeutic outcomes (5). Among several lifestyle modifications, adjustment of dietary salt intake is considered to favor outcomes in patients with T2D, obesity, and hypertension (6, 7).

Existing evidence lacks consistence concerning the impact of dietary salt intake on the targets of T2D treatment, such as blood glucose and glycated hemoglobin (HbA1c). A study reported that individuals with higher urine sodium excretion that reflects the amount of dietary salt intake had higher glucose levels than individuals with lower urine sodium excretion (6). Nevertheless, another study revealed a negative correlation between urine sodium excretion and fasting blood glucose (8), which might result from enhanced simultaneous reabsorption of glucose and sodium in the proximal tubule of the kidney due to increased sodium-dependent glucose transporter 2 (SGLT2) activity (9). Moreover, a meta-analysis concluded that a reduction in dietary salt intake did not affect fasting glucose in children or adults (10).

As T2D, kidney damage, and hypertension usually coexist in patients with T2D and dietary salt intake is positively correlated with blood pressure, most clinical guidelines recommend a reduced salt intake of 5–6 g NaCl/day for patients with T2D to alleviate diabetic complications associated with hypertension (11). The WHO guidelines reduce the daily dietary salt intake from 6 g to 5 g (12). Patients with T2D are advised to have optimized dietary salt intake besides glucose-lowering treatment. However, changing dietary habits is not easy for patients who are accustomed to a high salt diet. To what extent patients undergoing long-term T2D treatment are affected by dietary salt intake has not been completely investigated. Existing studies are primarily carried out among cross-sectional data that provide insufficient information about the impacts of dietary salt intake on glucose-lowering treatment. Moreover, a previous study reported that low salt diet increased insulin resistance in healthy subjects (13). Whether such an association exists in T2D patients also needs further investigation (14).

This study aimed to investigate the influence of dietary salt intake on T2D treatment, including glucose-lowering effect, insulin resistance, and indices related to T2D progression and complications. We classified patients into groups according to how they adhered to dietary salt optimization during diabetes treatment. We compared their one-year follow-up outcomes in terms of fasting blood glucose (FBG), glycated hemoglobin (HbA1c), blood pressure, obesity, and the prevalence of retinopathy and neuropathy among the groups.

## Methods

### Study Patients

This longitudinal observational study was conducted at Ningbo City First Hospital from January 1, 2018, to December 30, 2021 and registered on ClinicalTrials.gov (No. NCT03811470). Eligible patients were 18–70 years old and had T2D with HbA1c over 6.5% and FBG over 7 mmol/L. In total, 1090 patients were included and completed a sodium knowledge survey and a food frequency questionnaire (FFQ) for sodium intake (15). Their demographic features and lifestyle factors, physical examination, and laboratory tests were obtained at the first visit and referred to as baseline features (16). The follow-up survey of dietary habits was conducted monthly. After one-year treatment according to The American Diabetes Association (ADA) recommendations (17), the clinical features were compared with baseline features. The study was performed following the guidelines of the Helsinki Declaration and was approved by the Ethics Committee of Ningbo City First Hospital.

### Clinical Features

Each patient underwent a physical examination including measurements of height, weight, and systolic blood pressure (SBP). Body mass index (BMI) was calculated as weight (kilograms) divided by squared height (meters). Visceral f*at* area (VFA) was determined by multifrequency bioelectrical impedance (18). Fasting blood samples were obtained to measure FBG, HbA1c, triglycerides, total cholesterol, high-density lipid cholesterol (HDL-c), and low-density lipid cholesterol (LDL-c). 2-hour postprandial glucose (2h PBG) was measured 2 hours after diet. Albumin excretion in morning spot urine was calculated by urine albumin/creatinine ratio(UAR) (unit, mg/g) (19). Peripheral neuropathy was identified through electrophysiologic examination (Viasys Healthcare VikingQuest; Viasys Healthcare GmbH) of the right leg and distal motor latencies of the right tibial and peroneal nerves (20). Diabetic retinopathy was identified by an ophthalmologist using a digital nonmydriatic fundus camera (CR6-45NM; Canon, Lake Success, NY) (21). Primary outcomes after one-year treatment were the measures and their changes between baseline and follow-up time.

### Measurement of Dietary Salt Intake by FFQ

At the first visit and monthly after the beginning of the glucose-lowering treatment, the dietitian performed FFQ for all patients through 24-h dietary recalls (22). All foods, drinks, and dietary supplements consumed in the previous 24 h were recorded and classified. The patients were asked to identify discretionary salt use with teaspoon measures. According to FFQ results, the patients were required to rate their habitual daily salt intake as low, moderate, or high. Low was defined as <6 g/d, moderate as 6 to 8 g/d, and high as >8 g/d. Based on the dietary salt intake levels before and after treatment, the patients were classified into five groups: decreasing, increasing, steadily low, steadily medium, and steadily high dietary salt intake groups. The patients having decreasing dietary salt reduced their dietary salt intake during the treatment period compared to the baseline, whereas the increasing dietary salt patients increased their dietary salt intake. The patients having steadily low, steadily medium, and steadily high salt did not change their dietary salt habits before and during the treatment.

### Data Analysis

The data were analyzed using R statistical software (version 4.1). The normality of distribution of continuous variables was tested by one-sample Kolmogorov-Smirnov test. Normally distributed continuous variables were expressed as mean (standard deviation [SD]). Means of two continuous variables were compared using independent samples Student’s t test. Categorical variables were presented as percentages and compared among groups using Pearson χ2 or Fisher’s exact test. A value of *P* < 0.05 was considered significant.

## Results

A total of 1090 patients with T2D (350 [32.1%] women and 740 [67.9%] men; age, mean 48.4 and SD 11.7 years) were consecutively recruited from January 1, 2018, to December 30, 2021. The patients were classified into 5 groups according to dietary salt intake levels, including 287(26.3%) decreasing-, 190(17.4%) increasing-, 175(16.0%) steadily low-, 243(22.3%) steadily medium-, and 195(17.9%) steadily high-dietary salt intake patients (**Table 1**). The age of patients with steadily medium (50.4 ± 11.5 years) and steadily high salt (51.3 ± 10 years) was higher than the remaining three groups (all p<0.01), indicating that elder people tended to be more accustomed to a high salt diet. The prevalence of lowering glucose drugs among the patients was metformin (85%), dipeptidyl peptidase-4 (47.5%), glycosidase inhibitor (43.1%), sodium-glucose cotransporter 2 (SGLT-2) (41.5%), insulin (33.1%), sulfonylurea (32.2%), and glucagon-like peptide-1 receptor agonist (GLP-1) (29.9%) (**Table 1**). Except for a minor difference of metformin among the five groups (p=0.02), the remaining six drugs were used with similar frequency among the five groups (all p>0.05).

**Table 1:** Clinical features of patients with T2DM stratified by dietary salt intake.

Feature	All	Decreasing	Increasing	Steadily low	Steadily medium	Steadily high	*p*
Number	1090	287 (26.3%)	190 (17.4%)	175 (16.0%)	243 (22.3%)	195 (17.9%)	
Female	350 (32.1%)	96 (33.4%)	52 (27.4%)	67 (38.3%)	80 (32.9%)	55 (28.2%)	
Age (year)	48.4 ± 11.7	46.6 ± 12.1	47.4 ± 12.3	46.8 ± 11.7	50.4 ± 11.5	51.3 ± 10	<0.001
Metformin	923 (85%)	245 (85.4%)	160 (84.2%)	135 (77.1%)	208 (85.6%)	175 (89.7%)	0.02
Dipeptidyl peptidase-4	518 (47.5%)	149 (51.9%)	85 (44.7%)	82 (46.9%)	110 (45.3%)	92 (47.2%)	0.94
Glycosidase inhibitor	470 (43.1%)	133 (46.4%)	74 (38.9%)	81 (46.3%)	94 (38.7%)	88 (45.1%)	0.24
SGLT-2	452 (41.5%)	126 (43.9%)	70 (36.8%)	73 (41.7%)	94 (38.7%)	89 (45.6%)	0.34
Insulin	361 (33.1%)	103 (35.9%)	69 (36.3%)	49 (28%)	73 (30.0%)	67 (34.3%)	0.28
Sulfonylurea	351 (32.2%)	83 (28.9%)	58 (30.5%)	58 (33.1%)	81 (33.3%)	71 (36.4%)	0.48
GLP-1	326 (29.9%)	80 (27.9%)	54 (28.4%)	47 (26.9%)	77 (31.7%)	68 (34.8%)	0.38

**Figure 1:**
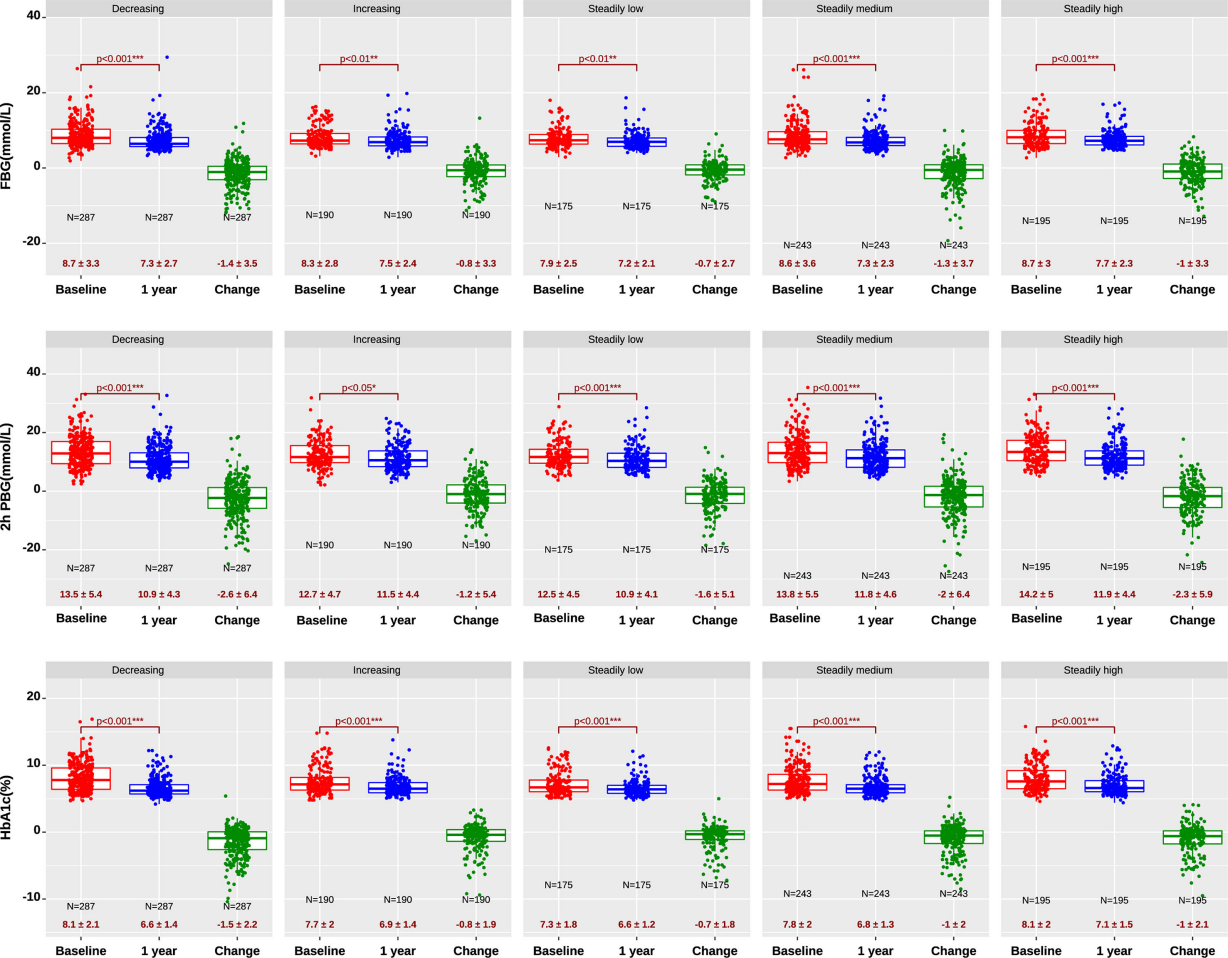
FBG (top), 2h PBG (mid), and HbA1c (bottom) at baseline and after one-year treatment. Decreasing and steadily low salt intake leads to lower baseline FBG, 2h PBG, and HbA1c, as well as a larger decrease in FBG, 2h PBG, and HbA1c after treatment. *, significant with p<0.05; **, significant with p<0.01; ***, significant with p<0.001.

At baseline, two groups of patients (decreasing and steadily high groups) with high salt intake had higher FBG (8.7 ± 3 mmol/L) than patients with steadily low salt intake (7.9 ± 2.5 mmol/L, p<0.01) (top of **Figure 1**). Overall, one-year treatment lowered mean FBG from 8.47 ± 3.12 mmol/L at baseline to 7.38 ± 2.4 mmol/L, with a mean decrease of 1.09 mmol/L. The largest decrease in FBG was 1.4 mmol/L in the decreasing salt group, significantly larger than that (0.8 mmol/L) in the increasing salt group (p<0.01). Briefly, high salt intake implicated higher baseline FBG, while reducing salt intake implicated a larger FBG decline during treatment. Similar changes were observed in 2h PBG (mid of **Figure 1**). Additionally, the baseline HbA1c and the change of HbA1c after one-year treatment resembled the relevant variations of FBG (bottom of **Figure 1**). The decrease in HbA1c in the decreasing salt group was 1.5% (SD, 2.2%) vs. 0.8% (SD, 1.9%) in the increasing and steadily high salt groups (p<0.01).

**Figure 2:**
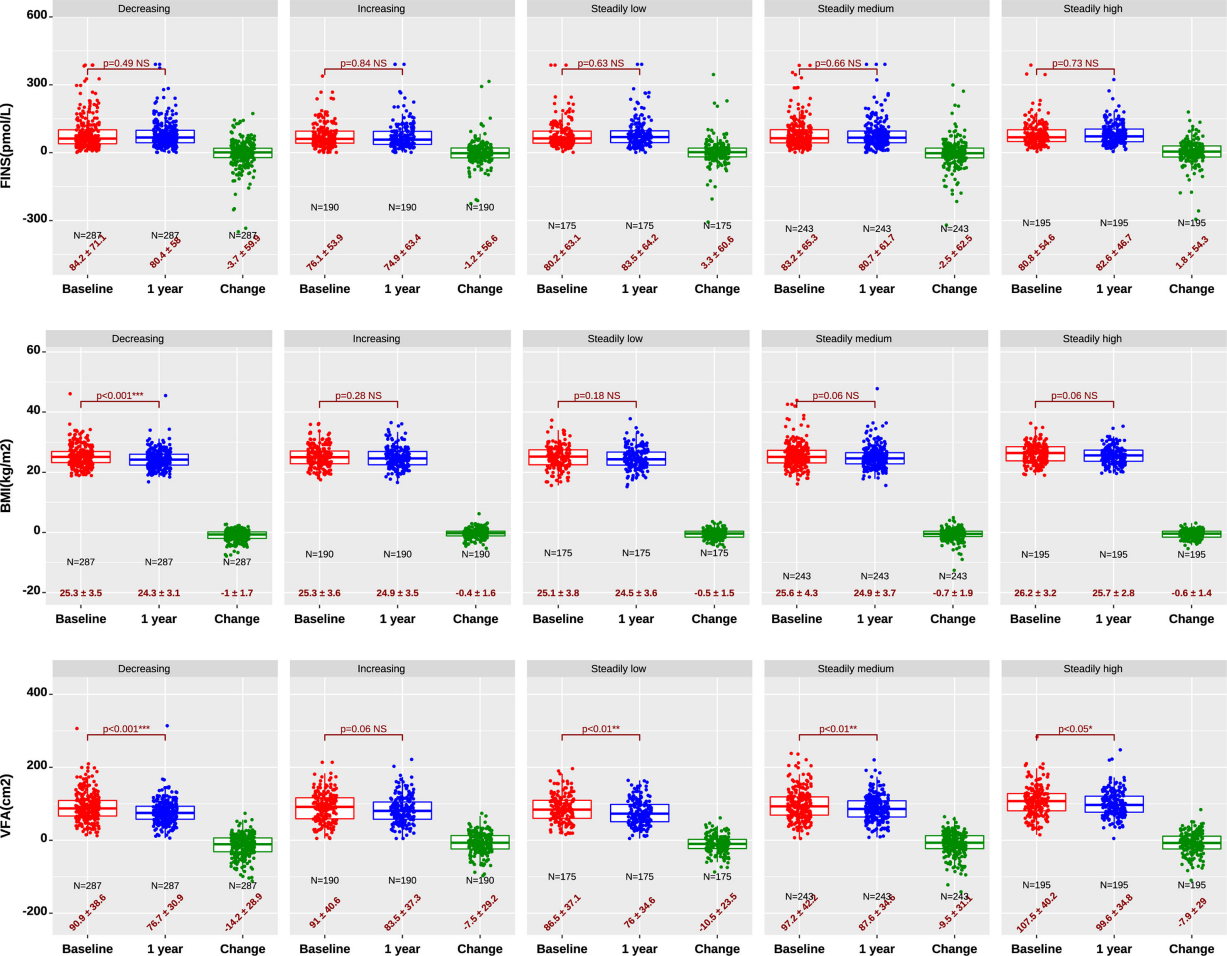
Fasting insulin(top), BMI(mid), and VFA (bottom) at baseline and after one-year treatment. The fasting insulin in the steadily low salt group showed a minor increase after treatment. Decreasing and steadily low salt intake leads to lower BMI and VFA, as well as a larger decrease in BMI and VFA after treatment. *, significant with p<0.05; **, significant with p<0.01; ***, significant with p<0.001; NS, not significant with p>0.05.

Regarding fasting insulin levels, there was no significant difference in all groups between baseline and one year (p>0.05) (top of **Figure 2**). Nevertheless, after one-year treatment, the fasting insulin in the steadily low salt group increased by 3.3 ± 60.6 pmol/L, which was significantly higher than the largest reduced fasting insulin of -3.7 ± 60 pmol/L in the decreasing salt group (top of **Figure 2**). This interesting finding suggested persistently low sodium might increase fasting insulin levels.

Notably, significant decrease of BMI during the follow-up was solely found in the decreasing salt intake group (p<0.01) (mid of **Figure 2**). Moreover, VFA was significantly decreased in four groups (p<0.05) except for the increasing salt intake group, with the decreasing salt intake group having the largest drop of 14.2 ± 28.9 cm^2^ (bottom of **Figure 2**).

**Figure 3:**
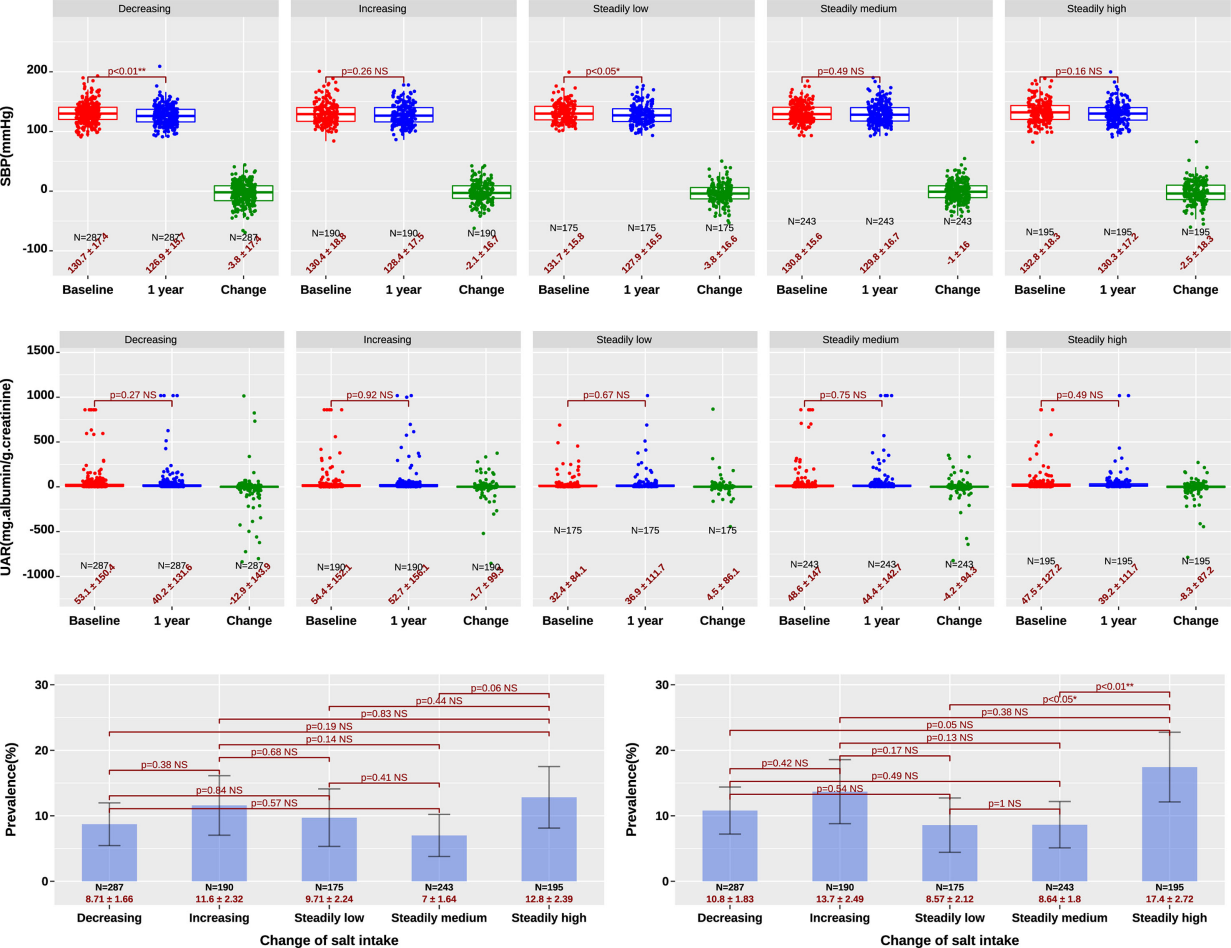
SBP (top), UAR (mid), and prevalence of retinopathy (left bottom) and neuropathy (right bottom). Decreasing and steadily low salt intake leads to lower baseline SBP and a larger decrease in SBP. The steadily low salt group had lower UAR both at baseline and after treatment. Decreasing and steadily low salt intake leads to a slightly lower prevalence of retinopathy and a significantly lower prevalence of neuropathy. *, significant with p<0.05; **, significant with p<0.01; NS, not significant with p>0.05.

The present study found that the decrease of SBP in the five groups ranged from 1 to 3.8 mmHg (top of **Figure 3**), with the largest decline (3.8 mmHg) in the decreasing and steadily low salt groups. As for the urine albumin/creatinine ratio (UAR) that reflected potential kidney impairment, the change after one-year treatment was not significant among the five groups (p>0.05), whereas the steadily low salt group had the lowest value both at baseline (32.4 ± 84.1 mg/g) and after treatment (36.9 ± 111.7 mg/g), which were significantly lower than the other four groups (p<0.01) (mid of **Figure 3**).

Concerning two major complications of T2D, the prevalence of retinopathy in the increasing and steadily high salt groups was 11.6%(95% CI, 7.1–16.1%) and 12.8%(95% CI, 8.1–17.5%), respectively, which were slightly higher than those in the remaining three groups, which were 8.71%, 9.71%, and 7% (the lowest p was 0.06) (left bottom of **Figure 3**), while the prevalence of neuropathy was 17.4% (95% CI, 12.1–22.7%) in the steadily high salt groups, which was significantly higher than that in the remaining three groups that were 10.8%, 8.57%, and 8.64% (all p<0.05), and 13.7% (95% CI, 8.8–18.6%) in the increasing high salt groups, which was slightly higher than the remaining three groups(p>0.05) (right bottom of **Figure 3**).

To summarize, patients with steadily high and increasing dietary salt had higher baseline FBG and BMI, lower reduction in FBG, VFA, and SBP after one-year treatment, as well as a slightly higher prevalence of retinopathy and a significantly higher prevalence of neuropathy. The steadily low salt group had lower UAR both at baseline and one-year treatment. Notably, the fasting insulin in the steadily low salt group showed a minor increase during treatment.

## Discussion

Our study revealed that dietary salt intake had benefits on a broad spectrum of T2D treatment targets, including glucose, HbA1c, as well as on SBP, BMI, VFA, retinopathy, neuropathy, and UAR. Patients who changed high dietary salt intake before treatment to low or medium salt intake significantly benefited glucose-lowering treatment. The study also suggested that persistently low salt intake might increase fasting insulin and subsequently increase insulin resistance.

The most important finding in the present study was that reduced dietary salt intake enhanced glucose-lowering effect in patients with high salt intake before treatment. The findings from the cross-sectional study exhibited that baseline FBG and HbA1c were higher in high dietary salt patients (p<0.05) (**Figure 1**), while the longitudinal one-year follow-up found that decreasing dietary salt contributed to a larger decrease in FBG and HbA1c, as compared to increasing and steadily high salt patients. These findings implicate that high dietary salt intake is associated with increased FBG and HbA1c and change to lower dietary salt benefits glucose-lowering treatment. Previous research mostly studied the influence of dietary salt intake on the main treatment targets of glucose and HbA1c using cross-sectional T2D cohorts, which yielded inconsistent conclusions with no relation to treatment effects. Part of previous research reported no evidence of decreased circulating glucose concentrations by large reductions in sodium (10), whereas other research concluded that high salt intake could promote T2D in mice (23). Additionally, there were studies that declared the elusive association between high dietary salt intake and high blood glucose (6, 8). We speculate that the inconclusive results about the impacts of dietary salt on blood glucose results from the different lengths of observation time and varied sample sizes when studying weak associations, as the previous conclusions were derived according to a short intervention of a large reduction in sodium, whereas our study compared baseline values with that after one-year treatment.

Another interesting finding here was that steadily low salt intake might increase fasting insulin and subsequently increase insulin resistance (p<0.01) (top of **Figure 2**). Similar phenomena were reported previously, indicating worsened insulin sensitivity in patients who underwent strict dietary sodium reduction, reporting that strict dietary sodium reduction to 30 mmol/day for a week increased plasma insulin by 40.6% without changing plasma glucose (13, 24). Although steadily low salt intake in our study did not reach such a low sodium level, we still observed that persistently having low salt intake might lead to increased insulin resistance. The effect of low sodium diet on insulin resistance is far more complicated to be disputable (14). We believe that the development of insulin resistance is a multiple-year-long progression, additional randomized controlled trials with an adequate study period and well-designed low sodium diet are needed to verify this finding.

The present study also revealed that the decreasing and steadily low salt intake patients had the largest reduction of SBP after one-year treatment (top of **Figure 3**). A notable point is that the steadily low salt intake patients had significantly lower UAR than the remaining four groups. This point suggests that beneficial outcomes for the kidney need a long time to achieve. The overall findings about SBP and UAR are consistent with previous research (25, 26).

High dietary salt intake is considered an independent risk factor of obesity (27). This conclusion was clearly seen in our study, as the increasing salt patients had a lower reduction of BMI than the decreasing salt patients (BMI decrease, 1.0 vs. 0.4, p<0.01) (mid of **Figure 2**). Noticeably, VFA that is closely associated with metabolic syndrome among T2D patients was positively linked to dietary salt intake levels both at baseline and one-year treatment time (bottom of **Figure 2**). This link seemed more distinctive than BMI, indicating VFA was a more sensitive index for evaluating impacts of dietary salt than overall BMI. To our knowledge, this finding is firstly identified and proves that reduced salt intake considerably lowers visceral fat composition, implicating a remarkable benefit of relieving metabolic syndrome through lowering dietary salt.

Finally, our study saw a slightly higher prevalence of retinopathy and a significantly higher prevalence of neuropathy in patients with increasing and steadily high salt (bottom of **Figure 3**). Such influence has been inconsistently concluded in a handful of previous studies. The influence on retinopathy and neuropathy observed in our study might result from hypertension and high blood glucose associated with a high salt diet (28, 29). Another explanation might be that a high-salt diet led to ischaemia/reperfusion-induced retinal neuronal impairment *via* activating pro-inflammatory and pro-apoptotic signaling pathways and inhibiting vasodilation (28).

This study had limitations. Several measures such as UAR and fasting insulin had great variations among patients, which might lead to biased conclusions. More studies with a larger sample are necessary to further investigate these measures.

## Conclusion

To summarize, the present study identifies that lowered dietary salt intake benefits T2D treatment in comprehensive aspects, as illustrated by a broad spectrum of reduced measures, including the main treatment targets such as FBG and HbA1c, and indices reflecting potential complications of T2D, including BMI, VFA, SBP, UAR, retinopathy and neuropathy. Our conclusions are insightful in the combination of drug therapy and optimization of dietary salt intake to achieve more beneficial treatment outcomes in patients with T2D.

## Data Availability Statement

The raw data supporting the conclusions of this article will be made available by the authors, without undue reservation.

## Ethics Statement

The study complies with the Declaration of Helsinki. The research protocol has been approved by the ethics committee of Ningbo City First Hospital. The patients/participants provided their written informed consent to participate in this study.

## Author Contributions

LL: design the study and contribute to the writing. YM and MX: collect data. JS and LR: review the manuscript. QS: analyze the data and write the manuscript. All authors contributed to the article and approved the submitted version.

## Funding

The Major Program of Social Development of Ningbo Science and Technology Bureau (Grant No. 2019C50094).

## Conflict of Interest

The authors declare that the research was conducted in the absence of any commercial or financial relationships that could be construed as a potential conflict of interest.

## Publisher’s Note

All claims expressed in this article are solely those of the authors and do not necessarily represent those of their affiliated organizations, or those of the publisher, the editors and the reviewers. Any product that may be evaluated in this article, or claim that may be made by its manufacturer, is not guaranteed or endorsed by the publisher.
